# Sex hormone-mediated change on muscle activation deactivation dynamics in young eumenorrheic women

**DOI:** 10.3389/fphys.2023.1104578

**Published:** 2023-03-07

**Authors:** Subaryani D. H. Soedirdjo, Luis A. Rodriguez, Yu-Chen Chung, Ellen Casey, Yasin Y. Dhaher

**Affiliations:** ^1^ Department of Physical Medicine and Rehabilitation, UT Southwestern Medical Center, Dallas, TX, United States; ^2^ Department of Bioengineering, University of Texas at Dallas, Richardson, TX, United States; ^3^ Department of Orthopedic Surgery, UT Southwestern Medical Center, Dallas, TX, United States; ^4^ Department of Physiatry, Hospital for Special Surgery, New York, NY, United States

**Keywords:** menstrual cycle, muscle activation deactivation dynamics, half relaxation time, time to peak torque, sex hormone

## Abstract

The goal of the study was to characterize muscle activation/deactivation dynamics across the menstrual cycle in healthy young women. Twenty-two healthy eumenorrheic women (age: 27.0 ± 4.4 years; mean ± SD) were tested every other day for one menstrual cycle. Serum estradiol and progesterone were quantified at the time of testing. Peak torque (PT), time to peak torque (TPT), and half relaxation time (HRT) of soleus muscle twitch were measured. Muscle twitch was elicited by delivering 1 ms width electrical pulses to the tibial nerve at an intensity that generated a maximum motor response (S-100) and at supramaximal intensity (S-120; 1.2 × S-100). The analyses were performed for each menstrual cycle phase: 1) the follicular phase to analyze the effect of estradiol while the progesterone concentrations remained at low concentrations; 2) the luteal phase to analyze the effect of progesterone with background estradiol concentrations. In the follicular phase, there was no association of estradiol for PT, TPT, and HRT. In the luteal phase, while estradiol had no association on PT, TPT, and HRT, progesterone expressed a significant association with HRT reduction but no association on PT or TPT. Also, there was a significant estradiol and progesterone interaction for HRT. However, the regression parameters are nearly zero, suggesting that the change in HRT may not have an impact on muscle performance across the menstrual cycle but implications on other women’s health conditions with elevated sex hormone concentrations, such as pregnancy, may prove critical.

## 1 Introduction

One of the key tenants of neuromuscular control is the muscle’s ability to generate a targeted force level over a specific period (time to peak force) and the time needed to deactivate the muscle (half relaxation time) in preparation for the next motor command required for a given movement (Neptune and Kautz, 2001). It has been shown that sex hormone receptors, such as estrogen and progesterone receptors, exist throughout the body including in the nervous system and skeletal muscles (Lemoine et al., 2003; Wiik et al., 2009; Gargiulo-Monachelli et al., 2014; Ekenros et al., 2017). Therefore, it is plausible that modulation in the estradiol and progesterone concentrations affect the neuromuscular function.

Estradiol and progesterone are two main sex hormones that naturally fluctuate in eumenorrheic women. During the follicular phase, the estradiol level increased from its minimum at menses to its peak before ovulation. It then decreases at ovulation, with a secondary estradiol peak during the luteal phase concomitant with the progesterone peak (Reed and Carr, 2000). While estrogen is known to have an important role in the regulation of muscle mass (Maltais et al., 2009), muscle metabolism (Hamadeh et al., 2005), and muscle regeneration (Velders and Diel, 2013), the effect of sex hormones on the basic motor control properties at the muscle level have not been fully delineated.

The mechanistic effect of sex hormones on skeletal muscle has been explored mostly using animal models. For example, estradiol-deprived animals (ovariectomized/OVX) expressed significantly shorter muscle HRT compared to controls (Horner and Lehmann, 1978; Fisher et al., 1998; Mccormick et al., 2004), which may be due to estradiol’s role as an inhibitor of sarcoplasmic reticulum Ca2+-ATPase (SERCA) (Khan, 1979; Martinez-Azorin et al., 1992). In addition, ovariectomized animals treated with estradiol showed a significantly longer half relaxation time (HRT) compared to OVX animals (Horner and Lehmann, 1978; Mccormick et al., 2004). On the other hand, isolated exposure to progesterone was shown to shorten the HRT (Horner and Lehmann, 1978; Moshal et al., 2014) which may partly correlate to an increase in sarcolipin expression (Price and Dai, 2015), a SERCA activity regulator. The opposing effects of progesterone and estradiol on SERCA activity suggest the presence of progesterone-estradiol interaction on the muscle relaxation process. Indeed, as reported in the lone study that we are aware of, the relaxation time of animals treated with estradiol and progesterone (OVX + E + P) is significantly longer compared to OVX animals (Horner and Lehmann, 1978).

Additionally, estradiol deprivation has been shown to induce a reduction in time to peak tension (Mccormick et al., 2004). It has been reported, however, that estradiol-deprived mice exhibit a lower peak tension (Collins et al., 2018), suggesting that estradiol has beneficial effects on force production, presumably due to the enhancement of muscle’s regulatory light chain phosphorylation (Stull et al., 2011; Lai and Lowe, 2013), an increase in the accessibility of actin-binding (Levine et al., 1998), or maintenance of muscle MyHC composition (Kadi et al., 2002). On the other hand, OVX animals treated with progesterone only had significantly lower specific tension (peak force/muscle mass) compared to OVX animals (Horner and Lehmann, 1978). Data in animal models on the effect of progesterone alone on time to peak torque is lacking. Examinations involving a combined treatment of estrogen and progesterone are scarce and show conflicting results. Combined estradiol-progesterone treatment on OVX animals showed no effect on peak tension compared to OVX animals (Horner and Lehmann, 1978). Schneider et al., however, reported that OVX + E + P animals have a significantly higher isometric peak force compared to OVX animals (Schneider et al., 2004). These conflicting results highlight the complexity of the interaction between progesterone and estrogen in the force production in animals.

Although animal studies suggest mechanistic pathways for the potential effects of estradiol and progesterone on muscle twitch characteristics, the estradiol and progesterone treatments are usually administered chronically (28 days) and at concentrations exceeding physiological concentrations reported in these animals (three to four times) (Hou et al., 2010; Ingberg et al., 2012; Ström et al., 2012). While informative, results from these examinations are difficult to interpret in the context of the current study which had an acute exposure of estradiol (measured in days) at substantially lower concentrations compared to the experimental animal model systems.

Assessing the effect of estradiol in humans is more straightforward when an examination is conducted in the first half of the menstrual cycle; a state with consistently low serum progesterone levels. While examining the isolated effect of progesterone may provide critical scientific insights, progesterone does not have an unopposed peak during the human menstrual cycle. The increase in progesterone in the second half of the menstrual cycle is combined with the secondary rise in estradiol. Thus, we utilized an experimental design to examine the effect of sex hormones on muscle characteristics by acquiring data every other day in the cycle.

The goal of our study was to evaluate the interplay between the fluctuation of sex hormones and muscle twitch properties (peak torque, time to peak torque, and relaxation time) across the menstrual cycle. Our examination was guided by evidence in animal models showing a change in muscle force production characteristics due to estradiol and progesterone treatments. For example, it has been shown that estradiol treatment significantly increases muscle relaxation time (Mccormick et al., 2004) while progesterone treatment significantly decreases muscle relaxation time (Moshal et al., 2014). Therefore, we hypothesized that an increase in estradiol level would lengthen the muscle’s time to peak torque and relaxation time. In addition, we hypothesized that an increase in progesterone level with background levels of estradiol would counteract the effect of estradiol.

## 2 Materials and methods

Healthy young eumenorrheic women from similar demographic groups were recruited for this study. Every-other-day testing sessions were conducted, and serum samples were acquired. A transcutaneous peripheral nerve stimulation paradigm was employed to mimic the afferent activation and deactivation of the muscle. Muscle contraction properties were compared between sessions to evaluate the effect of changes in estradiol and progesterone concentrations.

The experimental protocol used in this study was based on the H-reflex paradigm. The soleus muscle was selected as the model system due to the ease of eliciting the H-reflex compared to the gastrocnemius or rectus femoris, and to delineate responses that may incorporate activation of cutaneous pain receptor (Kameyama et al., 1989; Vieira et al., 2013). In addition, it has been established that the quality of H-reflex responses in the soleus muscle is better than the same responses in the gastrocnemius muscle due in part to the notion that the soleus has a higher density of muscle spindles, thus its motor neuron have greater tendencies to be recruited with the lower threshold (Tucker and Türker, 2004).

### 2.1 Subjects

Fifty-three healthy eumenorrheic females with moderate physical activity and no history of lower limb disorder were enrolled in the study. Women with moderate physical activity were defined as those who exercised less than seven hours per week and were not participating in competitive-level sports. We had to exclude a total of 31 women: two due to lack of adherence to the protocol, five due to sensitivity to electrical stimulation, three due to the absence of estradiol and/or progesterone fluctuations across the menstrual cycle, eight withdrew from the study, and 13 decided not to participate in any part of the study after being enrolled. Thus, the results presented here are based on data acquired from 22 women (age: 27.0 ± 4.4 years; BMI: 24.1 ± 3.5 kg/m^2^; cycle length: 30.4 ± 3.9 days; mean ± SD). All participants provided written informed consent. This study was approved by the University of Texas Southwestern Medical Center and the Northwestern University institutional review boards.

### 2.2 General procedures

Subjects participated in testing sessions every other day for one menstrual cycle. The first testing session was scheduled based on the subject’s preference, resulting in random starting days within the menstrual cycle across subjects. Testing sessions were conducted at a consistent time within the day to minimize the potential effect of diurnal fluctuation of hormone levels across visits per subject (Bao et al., 2003; Bungum et al., 2011). Participants were instructed to maintain stable exposure to caffeine, alcohol, and exercise for 12 h before each testing session.

**FIGURE 1:**
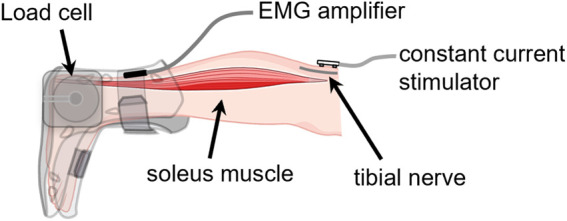
Muscle activation-deactivation testing configuration. Subject was in a prone position with their foot fixed in a boot coupled to a six-degrees-of-freedom load cell. The subject was relaxed while a series of electrical pulses were delivered to the tibial nerve at the popliteal fossa. Created partially with BioRender.com.

Each participant was placed in a prone position on a padded examination table with their knee fully extended and their ankle joint secured in a boot. The boot was affixed to a six degrees-of-freedom load cell (Figure 1). The ankle-boot-load cell complex was adjusted such that the lateral malleolus was aligned with the rotational axis of the load cell. The subject position was recorded and used in subsequent testing sessions. Subjects were asked to remain silent and motionless but were instructed to stay awake throughout the testing session.

### 2.3 Hormonal concentrations

**FIGURE 2:**
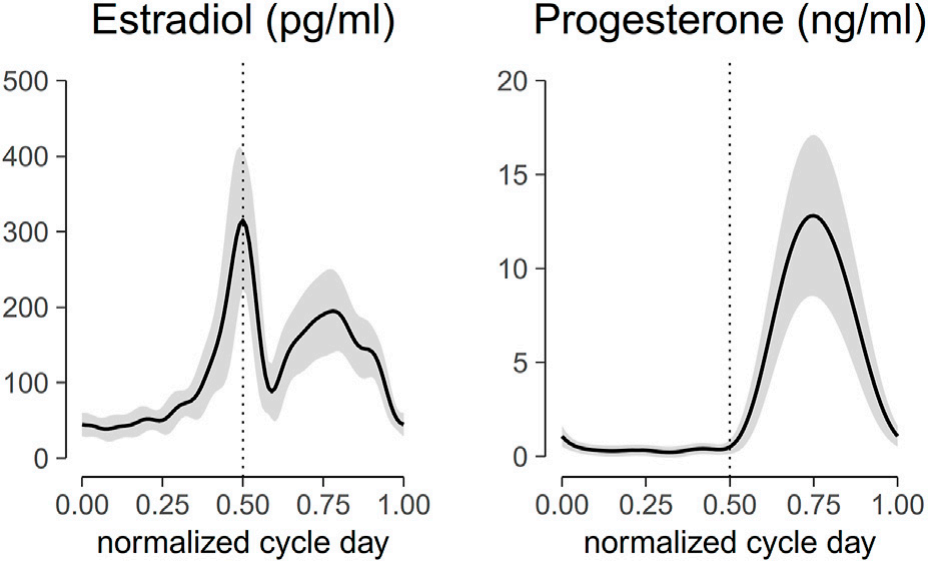
Aligned hormonal profiles from ovulatory cycles of the 22 subjects tested in the study. Serum was collected for measurement every other day for one cycle. The cycle day was normalized to cycle length and centered at peak estradiol concentration. Normalized cycle day 0 corresponds to the first day of menses, 0.5 corresponds to the peak of estradiol concentration, 0.75 corresponds to the peak of progesterone levels, and 1 corresponds to the last day of the cycle. The solid line represents the mean of all subjects, and the ribbon represents the standard deviation. Mid-cycle is marked with a dashed vertical line.

Venipuncture was performed on the antecubital area at each testing session using a 5 mL Vacutainer serum separator tube (BD, Franklin Lakes, New Jersey). Once the tube was filled, the sample was mixed well and allowed to clot. The samples obtained at Northwestern were processed by Northwestern Memorial Hospital Outpatient Laboratory and the samples obtained at UT Southwestern were sent to MedFusion. The blood samples were used to determine the serum concentrations of estradiol and progesterone. The cyclic patterns of these hormones over time were characterized using a non-linear mixed effect model with harmonics terms (Figure 2). The time was normalized such that the first day of menses was at time 0 and the end of the cycle was at time 1.0. Peak estradiol levels were centered at time 0.5 and the day of peak progesterone level was centered at time 0.75. This model offers an approach to investigating patterns accounting for within and across subject variations over time (Albert and Hunsberger, 2005; Mumford et al., 2012).

### 2.4 Surface EMG and torque recordings

Surface electromyography (sEMG) was performed using bipolar electrodes placed on the muscle bellies of the soleus (SOL) and the peroneus longus (PL). Two types of electrodes were used depending on the subject, either pre-amplified surface electrodes (DE-2.1 Bagnoli single differential, interelectrode distance: 10 mm, contact dimensions 10 × 1 mm) or bipolar Ag/AgCl pre-gelled electrodes (diameter 10 mm, inter-electrode distance 2 cm). The sEMG electrode type used in a given subject was consistent throughout the testing sessions. Before electrode placement, the skin was shaved, lightly abraded using Nuprep® (Weaver and Company, CO, United States), and cleansed with water to remove any residue. Each electrode site was measured with respect to bony landmarks and marked with a permanent marker to ensure the exact electrode placement at subsequent testing sessions. Subjects were encouraged not to wash off the markings between sessions. The ankle torque was measured using a six degrees-of-freedom load cell (JR3®, CA, United States) attached to a boot and concentric with the axis of the ankle. Both the EMG and load cell data were amplified (Micro1401 Data Acquisition Unit, Cambridge Electronic Design, United Kingdom) and sampled at 2,000 Hz.

### 2.5 Electrical stimulation

**FIGURE 3:**
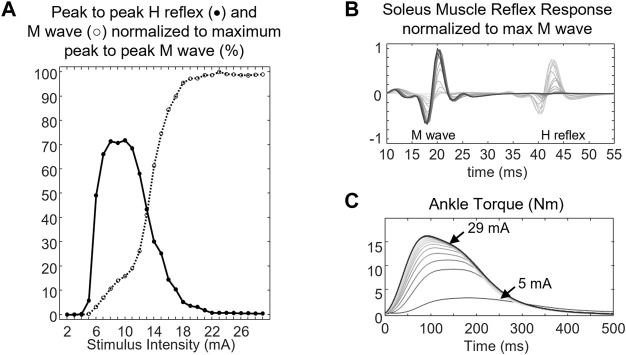
**(A)** peak-to-peak H reflex (closed circle, •) and M wave profile (open circle, ○), **(B)** soleus muscle response, and **(C)** ankle torque of a representative subject. The ankle torque increased as the stimulus intensity delivered to the tibial nerve increased. Lines with darker colors denote muscle response and ankle torque at higher stimulus intensity.

The motor response (M-wave) and H-reflex of SOL muscle were evoked by delivering a 1-ms square pulse from a DS7A constant current stimulator (Digitimer Ltd., Herfordshire, England). The location of the bipolar stimulating electrode (electrode diameter 0.8 cm, inter-electrode distance 2 cm) on the tibial nerve was optimized by moving the stimulating electrode over the nerve until a low-current stimulus elicited the H-reflex. The electrode placement was fixed with a medical tape after a minimal response was observed in the PL muscle while significant SOL muscle reflex response was recorded. The recording started by progressively increasing the stimulus intensity in 1-mA increments starting from 1 mA and continued until the peak-to-peak M-wave had reached a plateau over at least five trials (Figure 3B). The stimuli were delivered at 10- to 20-s random intervals to prevent post-activation depression (Hultborn et al., 1996). The stimulus intensity that generated the maximal peak-to-peak M-wave was considered the maximum stimulus intensity (S-100). Five measurements of the maximum M-wave at 10–20 s intervals were obtained. To compute the associated changes in the contractile properties of the muscle, five stimuli at supramaximal intensity (S-120: 120% of stimulus intensity needed to evoke maximal peak-to-peak M wave) were delivered to the tibial nerve at 10–20 s intervals (Grosset et al., 2005).

### 2.6 Signal analysis

The torque data obtained from the load cell were processed offline using Matlab software (R2019a, MathWorks Inc., MA, United States). A fourth-order zero-lag Butterworth low pass filter with a final cut-off frequency of 10 Hz was applied to the torque signal.

### 2.7 Muscle contractile properties

**FIGURE 4:**
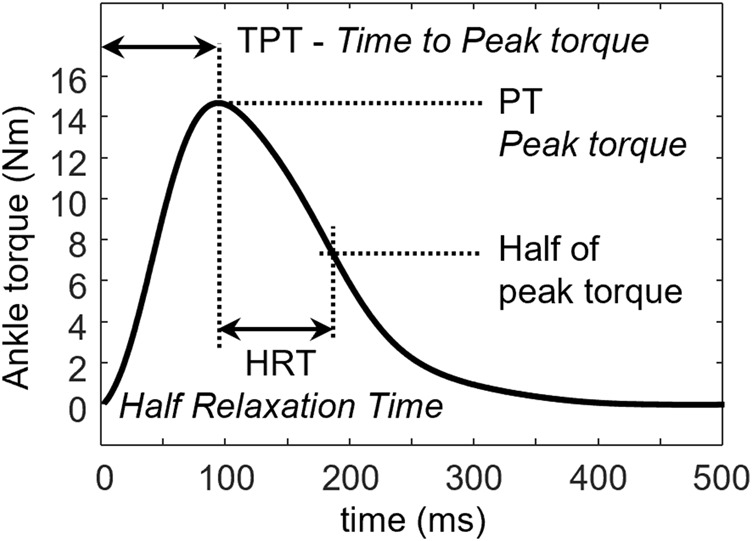
Half relaxation time (HRT) is defined as the time needed by the muscle to relax from the peak torque (PT) to 50% of its peak value. Time to peak torque (TPT) is defined as the time from the onset of the torque to PT.

Peak torque (PT), time to peak torque (TPT), and half relaxation time (HRT) were measured from the twitch evoked at the maximum stimulus intensity (S-100) and the supramaximal intensity (S-120) (Figure 4). TPT was calculated as the time from the onset of ankle torque to the PT. HRT was defined as the time needed for the muscle to decrease its force to 50% of its maximum.

The twitch response was analyzed at 1) the follicular phase where the estradiol level increased from a low level to its maximum while the progesterone levels remained low, and 2) the luteal phase where the progesterone increased from a low level to its maximum along with corresponding changes in the background estradiol levels.

### 2.8 Statistical analysis

Data are presented as mean ± SD. The mean values of PT, TPT, and HRT obtained from each subject were used for statistical analyses. The statistical analysis for this study was performed in the R language (version 4.2.2). A mixed-effect model two-way ANOVA was used to analyze the effect of menstrual phase and stimulus intensity on the peak torque (PT) of the muscle. To evaluate the effect of changes in hormone concentrations in the follicular and luteal phases on TPT and HRT, a generalized estimating equation (GEE) was employed using the geepack R package (Ballinger, 2004; Halekoh et al., 2006). Significance was accepted for *p* values <0.05. A GEE approach is typically used to develop population average models because it take into account the correlation of within subject data which may violate the independence assumptions made by traditional regression procedure (Hubbard et al., 2010).

## 3 Results

### 3.1 Hormone profile

The estradiol and progesterone profiles obtained from a mixed-effect model with harmonic terms are shown in Figure 2. The cycle day was normalized to the cycle length and centered such that 0 corresponds to the first day of menses, 0.5 corresponds to peak estradiol, 0.75 corresponds to peak progesterone, and 1.0 corresponds to the last day of the cycle. Estradiol concentrations on the first day of menses, peri-ovulatory (peak estradiol concentration), and mid-luteal (peak progesterone concentration) were 44.7 ± 15.4 pg/ml, 315.2 ± 92.4 pg/ml, and 190.3 ± 55.9 pg/ml, respectively. The corresponding progesterone concentrations at those time points were 1.1 ± 0.6 ng/ml, 0.5 ± 0.4 ng/ml, and 12.8 ± 4.3 ng/ml. These concentrations are consistent within the range of estradiol and progesterone concentrations reported in the literature (Sehested, 2000; Marsh et al., 2011; Mumford et al., 2012).

### 3.2 Peak torque (PT) at maximum stimulus intensity (S-100) and supramaximal stimulus (S-120)

**FIGURE 5:**
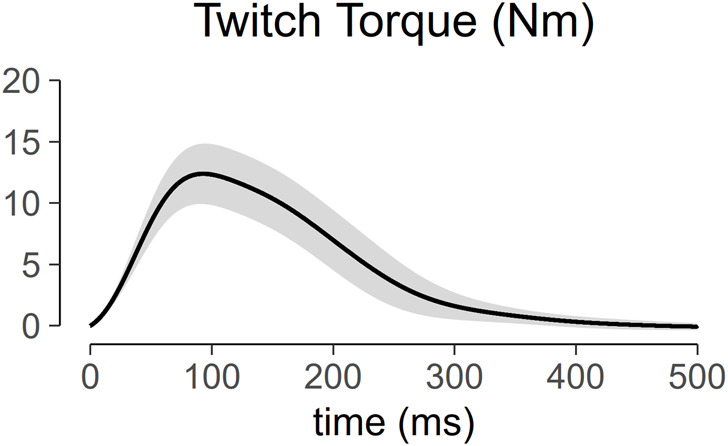
Profile of muscle twitch torque generated at maximum stimulus intensity (S-100). The solid line represents the mean of the 22 subjects, and the ribbon represents the standard deviation.

**FIGURE 6:**
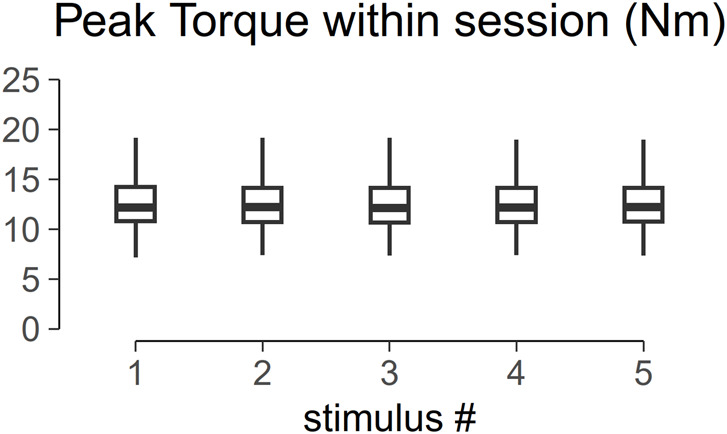
Box plot of the peak torque (PT) within one session. Analysis of variance shows no significant difference across trial within one session (*p* > 0.05).

Figure 5 shows the torque profile of the muscle twitch elicited at S-100 of 22 subjects across the menstrual cycle. Analysis of variance of PT within one session is shown in Figure 6. PT collected in two consecutive sessions during menses, at equivalent endocrine states, shows no significant differences (Table 1).

**TABLE 1:** Peak torque and estradiol concentrations collected in two sessions during menses.

	Menses 1	Menses 2	*p-value*
Peak torque (Nm)	12.3 ± 2.6	12.3 ± 2.7	0.80
Estradiol (pg/ml)	34.9 ± 11.5	41.5 ± 14.8	0.20

**FIGURE 7:**
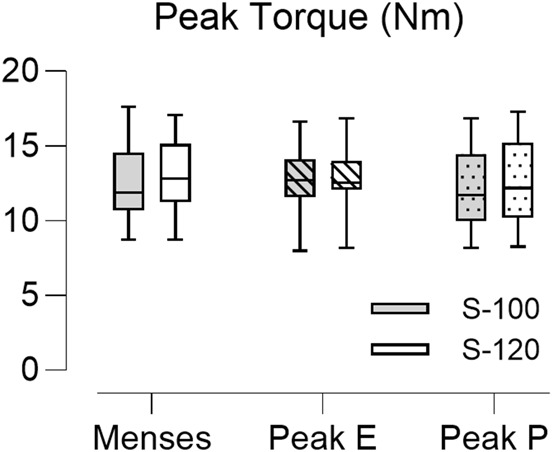
Boxplot of the peak torque (PT) obtained from 22 subjects across the three key time points in the menstrual cycle. Peak E indicates sessions at peak estradiol level and Peak P indicates sessions at peak progesterone level. The closed bar represents data obtained at maximum stimulus intensity (S-100) and the open bar represents data obtained at supramaximal stimulus intensity (S-120). Stimulus intensity and menstrual phase did not associate with PT values (*p* = 0.664 and *p* = 0.481 respectively).

To evaluate the effect of stimulus intensity on the degree of muscle recruitment across the menstrual cycle, the PT obtained at three key time points (menses, peak estradiol, and peak progesterone) were compared. The values did not differ between the sessions at menses, peak estradiol level (peak E), and peak progesterone level (peak P) (Figure 7). There was no significant difference between PT elicited at S-100 and S-120, suggesting that most of the available motor units were recruited at the S-100 stimulus intensity and the aggregate recruitment of these motor units did not change the net force production across the different visits.

### 3.3 Muscle twitch properties at maximum stimulus intensity (S-100)

The increase in estradiol concentration in the follicular phase of the menstrual cycle (normalized day 0 to day 0.5) was not associated with either TPT or HRT (Table 2, *p* > 0.05). In the luteal phase (normalized day 0.5 to day 1), TPT was not associated with the fluctuation of either estradiol or progesterone. As indicated in Table 2, GEE revealed a main effect of progesterone (*b* = −0.007, *p-value* = 0.006) and estradiol × progesterone interaction (*b* = 4.7 × 10^−5^, *p-value* = 0.002) on HRT. Our data suggest that an increase in progesterone in the luteal phase predicts decreases in HRT better than chance depending on the concentration of estradiol.

**TABLE 2:** Generalized estimating equation (GEE) of the main effect of estradiol, progesterone, and estradiol × progesterone interaction on peak torque, time to peak torque, and half relaxation time of muscle twitch across the menstrual cycle. The data were analyzed separately within follicular phase (estradiol concentrations increase to its maximum while the progesterone concentrations were low) and luteal phase (progesterone concentration increased concomitantly with estradiol).

Variables	GEE, *b* (*p*-value)		
	Estradiol (pg/ml)	Progesterone (ng/ml)	Interaction
Follicular phase			
Time to peak torque (ms)	−9.02 × 10^−5^ (0.072)	−	-
Half relaxation time (ms)	6.50 × 10^−5^ (0.417)	−	−
Luteal phase			
Time to peak torque (ms)	5.13 × 10^−4^ (0.227)	3.03 × 10^−3^ (0.219)	−3.62 × 10^−5^ (0.126)
Half relaxation time (ms)	−4.90 × 10^−4^ (0.072)	−6.94 × 10^−3^ (0.006) *	4.74 × 10^−5^ (0.002) *

## 4 Discussion

The goal of this study was to determine if the aggregate muscle twitch characteristics (peak torque, time to peak torque, and half relaxation time) modulate with fluctuating sex hormones throughout the menstrual cycle in young healthy women.

Our primary hypothesis was that the time to peak torque and muscle half relaxation time would increase with peak estradiol concentration during the periovulatory phase. We further hypothesized that progesterone would inhibit the effect of estradiol. Our results show that peak torque as well as time to peak torque did not change across the menstrual cycle. In the luteal phase, on the other hand, it was indicated that an increase in progesterone concentration was associated with a reduction in half relaxation time and the effect was modulated by the concentration of estradiol. However, the regression parameters indicate that the associations may be negligible.

Compared to animal studies (Horner and Lehmann, 1978; Mccormick et al., 2004; Schneider et al., 2004; Collins et al., 2018), the results of this study indicated that estradiol concentration was not associated with PT, TPT, or HRT. On the other hand, in the luteal phase, progesterone concentration was associated with shorter HRT depending on the estradiol concentration, however with low effect.

### 4.1 Increase of estradiol and muscle twitch properties during the follicular phase

Evidence in the literature regarding estradiol-mediated changes in muscle twitch characteristics in humans is scarce. Sarwar and colleagues found that the half relaxation time of the quadriceps muscle increased by up to 20% at the peri-ovulatory phase compared to menses, while Janse De Jonge and Kubo demonstrated that muscle twitch characteristics across the menstrual cycle were invariant (Sarwar et al., 1996; Janse De Jonge et al., 2001; Kubo et al., 2009). These conflicting results may be attributed to the low-resolution testing over the menstrual cycle and the potential differences in the relative levels of progesterone and estradiol during the luteal phase between the two studies. We argue that our every other day study design has a higher likelihood to capture the relationship between sex hormones and muscle twitch characteristics.

We found that an increase in estradiol over a short period (10–14 days exposure) expressed no association with soleus muscle peak force, and activation and de-activation dynamics. These findings are consistent with results from previous studies of the soleus muscle (Kubo et al., 2009) but not the quadriceps muscle (Sarwar et al., 1996). One distinguishing feature between the soleus and the quadriceps muscle is in their fiber composition. The soleus is primarily composed of type I fibers while the quadriceps muscles have a higher component of type II fibers (Edgerton et al., 1975; Staron et al., 2000). In rats, it has been suggested that estradiol may exert a differential effect on muscles with different fiber compositions due to a higher estrogen receptor (ER) content in type I fibers (Lemoine et al., 2002; Brown et al., 2009). While it is unlikely that the discrepancy in relaxation times between Sarwar et al., Janse De Jonge et al., Kubo et al., and our study are related to estradiol’s classical genomic pathway, it is still plausible that estradiol could have a differential effect on type II fibers *via* an ER-independent mechanism. While the exact mechanism is unknown, it could be suggested that estradiol targets the calcium handling of the fiber *via* the sarcoplasmic reticulum, which is the primary driver of the relaxation of the muscle (Harridge et al., 1996), and may explain the difference in relaxation times over the menstrual cycle between the quadriceps muscles and the soleus.

In our paradigm, during the activation phase of the twitch, the observed torque at the joint level is an aggregate effect of the muscle and the tendon. Tendon, a collagenous tissue, is affected by the change in estradiol concentrations in animal models (Lee et al., 2004), but similar data in humans is limited. Several examinations on the effect of changing estradiol on the laxity of similar collagenous connective tissue have been reported in humans. For example, Casey et al. reported that the anterior knee laxity in eumenorrheic women was not significantly different throughout the menstrual cycle, suggesting that collagen-based connective tissue mechanics were not modulated with changes in systemic levels of estradiol (Casey et al., 2014). Burgess et al. observed that the length, cross-sectional area, and stiffness of the Achilles tendon did not change throughout the menstrual cycle (Burgess et al., 2009). On the other hand, several other studies have shown that anterior knee laxity was modulated at different phases of the menstrual cycle (Shultz et al., 2004; Zazulak et al., 2006). Taken together, these findings suggest that the inconsistency in the literature on the effect of estradiol on musculotendon twitch properties may have origins in both the tendon and muscle components.

### 4.2 Fluctuation of progesterone, estradiol, and muscle twitch properties during the luteal phase

The impact of progesterone on muscle twitch characteristics remains inconclusive. In agreement with Kubo et al., 2009, our data indicated that during the luteal phase, fluctuation of estradiol concomitant with progesterone (in a physiologically relevant state) was not associated with any change in the muscle peak torque, time to peak torque, and half relaxation time (GEE regression parameters was close to zero). These findings are in direct contrast with the prevailing hypothesis that progesterone, through its vascular effects (White et al., 1995), may alter muscle force-generating capacity (Yasuda et al., 2009). Further studies examining the effect of isolated progesterone treatment, as well as combined estrogen-progesterone treatment, on muscle force-generating capacity are warranted.

We hypothesized that following progesterone treatment, HRT would shorten. In humans, high-dose progesterone (100–200 mg/day for 10–20 weeks) has been used as a treatment to prevent pre-term labor (Norwitz and Caughey, 2011) by inducing muscle relaxation through the production of nitric oxide (Renzo et al., 2005). It is important to note that during these interventions, recipients additionally express systemically high estradiol levels due to pregnancy (Soldin et al., 2005). To what extent the clinically observed effect of progesterone in this cohort is mediated or synergistically influenced by the background estradiol levels remains unknown. Our data indicate that during the normal co-fluctuation of the two hormones in the luteal phase, the effect of progesterone seems to be dependent on the estradiol concentration; a finding that highlights the complex interactions between the two hormones and warrants further examination.

### 4.3 The acute increase in sex hormones and rate of injury

In recent years, there has been increasing interest in tailoring the female athletic training regime around their menstrual cycle (Bruinvels et al., 2022) to optimize the athlete’s performance. We argue that the menstrual cycle-specific phase training paradigm is premature given the conflicting evidence linking cycle state and injury (McNulty et al., 2020; Nédélec et al., 2021). Evidence exists that the risk of joint injury is greater in the late follicular phase, a state of increased concentration of estradiol. Contrary to what we expected, our results demonstrated that the acute change in estradiol concentration during ovulation does not affect muscle twitch properties, a basic unit in muscle recruitment and function at the joint level. This inconsistency suggests that the increase in the frequency of injury around ovulation may not be attributed to the effect of estradiol on muscle but may be attributed to the estradiol-modulated effect on the neural state (Smith and Woolley, 2004). One can also observe that the reduction in the rate of injury in the second half of the cycle cannot exclude the potential effect of sex hormones. Our data did show that changes in half-relaxation time were associated with the combined change in progesterone and estradiol in the luteal phase. However, the hormone-associated changes may be minimal.

### 4.4 Monitoring neuromuscular adaptation and electrical stimulation-induced muscle damage

One could argue that an unaccustomed bout of exercise or stimulation can result in muscle damage, and this damage could alter the muscle twitch properties, thus, confounding our results. In the literature, it has been shown that electrical stimulation of muscle can significantly increase serum creatine kinase concentrations, an indirect measure of muscle damage (Aldayel et al., 2010; Vanderthommen et al., 2012). However, these protocols utilized 5 to 6-second-long contractions at 40%–100% of the maximum force. In this study, there were a total of 5 stimuli, each electrical stimulus has 1 ms duration, and the interstimulus interval was 10–15 s. For each stimulus, the twitch contraction period was about 200 ms (Figure 5). Our within session data showed an insignificant difference between the first and the fifth stimuli with the largest change of PT within subjects being 1.25 Nm. Thus, there was no within session electrical stimulation induced fatigue. In addition, changes in ankle torque during maximal voluntary contraction across all visits were also not significant. Taken together, these findings suggest the absence of potential neuromuscular adaptations or muscle damage within and across visits.

### 4.5 Limitations

The present study investigated the possible association between the fluctuation of estradiol and progesterone and muscle twitch characteristics. The data we collected and our ability to interpret the findings are not without limitations, including a small sample size. An additional uncontrolled factor is a possibility that muscle twitch characteristics are also dependent on a complex interaction with other hormones, such as testosterone (Prezant et al., 1993; Hirschberg et al., 2020) and relaxin (Dehghan et al., 2014), factors that were not incorporated in the present design. Future investigations should also include the measurement of muscle damage-associated biomarkers such as creatine kinase to help monitor any potential muscle damage that may be caused by an unaccustomed bout of exercise or stimulation.

### 4.6 Conclusion

This is the first study to use an every other day testing paradigm to investigate the sex hormone-mediated change of muscle contraction properties. This testing paradigm allowed for the analysis of the effect of changes in estradiol levels and the combined effect of progesterone and estradiol levels on musculotendinous properties. Further exploration is necessary to unfold the mechanism of interaction between elevated concentrations of estradiol and progesterone and muscle activation and deactivation dynamics.

## Data Availability

The raw data supporting the conclusion of this article will be made available by the authors, without undue reservation.
